# Cancer vaccine trial evaluations: immunobridging and potential immunological endpoints

**DOI:** 10.1093/immadv/ltaf016

**Published:** 2025-04-20

**Authors:** Ahmed Hussain, Benjamin Moxley-Wyles, Michael Bryan, Alex Gordon-Weeks, Ibrahem Al-Obaidi, Ciaran Sandhu, Lennard Lee

**Affiliations:** Medical Sciences Division, University of Oxford, Oxford, United Kingdom; Department of Cellular Pathology, Oxford University Hospitals NHS Foundation Trust, University of Oxford, Oxford, United Kingdom; Nuffield Department of Medicine, University of Oxford, Oxford, United Kingdom; Nuffield Department of Surgical Sciences, University of Oxford, Oxford, United Kingdom; Medical Sciences Division, University of Oxford, Oxford, United Kingdom; Medical Sciences Division, University of Oxford, Oxford, United Kingdom; Nuffield Department of Medicine, University of Oxford, Oxford, United Kingdom

**Keywords:** cancer vaccines, immunobridging, clinical trials, immunological surrogate, trial design

## Abstract

Therapeutic cancer vaccines are an emerging class of immunotherapy, but challenges remain in effectively adapting approved vaccines to a growing number of adjuvants, combination therapies, and antigen-selection methods. Phase III clinical trials remain the gold standard in determining clinical benefit, but are slow and resource intensive, whilst radiological surrogates often fail to reliably predict clinical benefit. Using immunological surrogates of efficacy, deployed in ‘immunobridging trials’, could present a viable alternative, safely speeding up cancer vaccine development in a cost-effective manner. Whilst this approach has proven successful in infectious disease vaccines, identifying reliable immunological correlates of protection has proven difficult for cancer vaccines. Most clinical trials, which present the richest source of data to establish a correlate, rely on peripheral blood samples and standard immunoassays that are ill-equipped to capture the complexity of the vaccine-induced anti-tumour response. This review is the first to outline the importance and challenges of establishing immunological surrogates for cancer vaccines in the context of immunobridging trials, evaluating current immunoassay methods, and highlighting the need for techniques that can characterize tumour-infiltrating lymphocytes and the suppressive tumour microenvironment across a range of patients. The authors propose adapting trial designs for surrogate discovery, including combining phase I/II trials and the use of multi-omics approaches. Successful immunological surrogate development could enable future immunobridging trials to accelerate the optimization of approved cancer vaccines without requiring new phase III trials, promoting faster clinical implementation of scientific advances and patient benefits.

## Introduction

Immunotherapeutic cancer vaccines aim to selectively target and destroy cancer cells. This is done by activating T and B-cells to target tumour-associated antigens (TAAs) or tumour-specific antigens (TSAs) presented on the surface of tumours. Despite the failure of several high-profile phase III clinical trials [1, 2], there has been renewed interest in the development of these vaccines. Over 360 cancer vaccine candidates are in active trial [3], and the recent breakthrough therapy designation of Moderna and Merck’s mRNA-157-P201 vaccine [4] against melanoma by the FDA, suggests that therapeutic cancer vaccines could be a pivotal new development against cancer.

**Figure 1.**
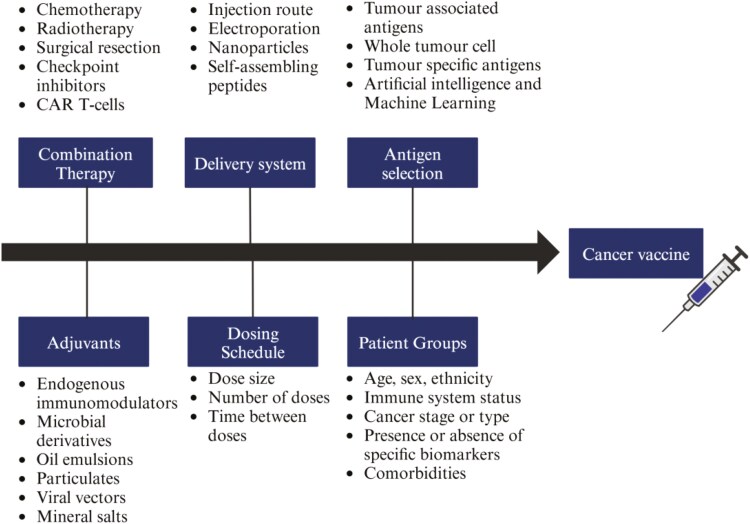
Sources of variability. Changes in each category may detract from or contribute to the efficacy of a given vaccine candidate.

Challenges remain in ensuring the fast and safe approval of cancer vaccines by regulatory authorities [5]. Randomized phase III clinical trials are the gold standard for measuring clinical endpoints. However, they tend to be expensive [6] and time-consuming. They are also poorly adapted at evaluating different combinations of therapies [7], delivery platforms, antigen-selection methodologies and adjuvants [8] (Fig. 1), all of which may contribute to or detract from the efficacy of a given vaccine. Radiological surrogate markers based on tumour size, which are used to accelerate the approval of conventional cytotoxic therapies, are often poorly suited for evaluating immunotherapies [9]. Cancer vaccines require a longer period of time to induce radiological changes and in the short term, can increase the size of tumours due to immune cell infiltration, giving the appearance of growth [10].

**Figure 2.**
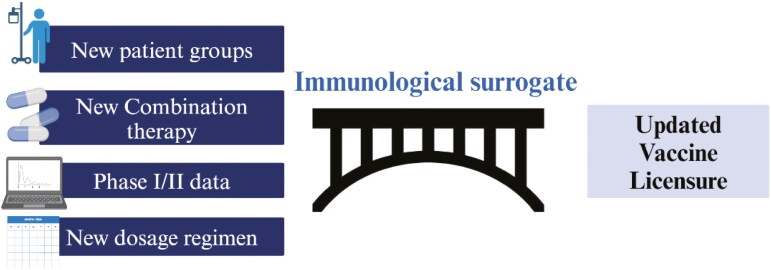
Example applications of immunobridging trials.

A yet unutilized method to overcome these challenges is to use the immune response elicited by cancer vaccines as a surrogate marker for clinical efficacy. This has parallels with vaccine development for infectious disease, where ‘immunobridging trials’ are deployed [11, 12]. In these trials, instead of using clinical endpoints, a validated immunological surrogate, known as a correlate of protection (CoP) [13], is used to infer efficacy based on immunological biomarkers measured in patients. For cancer vaccines, creating and utilizing validated immunological surrogates can help to ascertain whether a vaccine candidate is likely to be effective or not before any observation of clinical benefit or radiological change [14]. This can then provide evidence to support ‘marketing approval’, continuously improve and optimize approved vaccines according to new combinations of treatments and technologies, and support application to new patient cohorts [15] (Fig. 2). Additionally, the use of immunological surrogates can enable adaptive cancer vaccine trial designs, where patient dosing, likely clinical response and combination therapies can be evaluated in real time based on each patient’s immune response. Immunobridging trials are likely to become increasingly important as advances in precision medicine make it difficult to recruit large groups of patients and adequately power trials. This can also provide guidance to regulatory bodies in approving changes based on the development of novel technologies, such as changes made to AI algorithms used in neoantigen selection and optimization [16]. This would reduce the need to repeat costly and time-consuming phase III trials, without compromising on safety or clinical efficacy (Table 1).

**Table 1.** Comparison between typical immunobridging trial and phase III clinical trials.

Category	Immunobridging trial	Phase III clinical trial
Number of participants	100s of participants. Smaller trial size due to shorter time horizon and fewer treatment arms.	100–1000s of participants.
Endpoint	Immunological surrogate endpoint (e.g. T-cell response)	Clinical endpoint with long-term follow-up (e.g. Overall Survival)
Time to results	0–1 year	1–3 years
Cost	£1–5 million	£10–25 million

This review is the first to examine the application of immunobridging to therapeutic cancer vaccines. As such this review will focus on:

1) Outlining a potential framework for the application of immunobridging in the context of cancer vaccines.
2) Identifying and overcoming potential challenges associated with forming immunological surrogates.
3) Suggesting a set of recommendations for the design of current and future clinical trials.

## Cancer vaccine immunobridging

Immunological endpoints are biological measures that assess the ability of a therapy, such as cancer vaccines, to elicit an immune response. These measurements may closely correlate with clinical outcomes, such as preventing cancer-related death or inducing disease remission. If this correlation is established, immunobridging trials could become a widely accepted form of clinical trial, as immunological responses would serve as surrogate markers for clinical efficacy. For cancer vaccines, these endpoints would be derived using immunological data collected via immunoassays on patients before, and at various points after, vaccination [17]. This means most applications of immunobridging would occur post-approval of a therapeutic vaccine, once licencing trials have occurred, and adequate clinical data has been collected to validate an immunological surrogate [12, 18].

However, historically, cancer vaccine trials have failed to establish validated immunological surrogates. This is in part due to a lack of standardized immunoassays that can sufficiently account for the complexity of the anti-tumour immune response [19]. Current trials are also poorly adapted for the consistent collection of immunological data from patients. For instance, different clinical trials for the same vaccine candidate use different measures of the immunological response or different assays, making cross-comparison and pooling of data challenging [20]. Immunological markers tend to be evaluated for their predictive capacity rather than as surrogates, with often only baseline patient immunological data being characterized. Failure to optimize clinical trial data to adequately form immunological surrogates may hinder the adoption of immunobridging trials in future, slowing the development and updating of cancer vaccines in later-stage trials and post-approval.

Although no major regulatory body has yet to approve the use of an immunological surrogate for cancer vaccines, it has licenced infectious disease vaccines based on immunological markers, provided they are ‘reasonably likely’ to predict clinical benefit [18]. However, compared to infectious disease vaccines, the immune response for cancer vaccines may be more complex. For infectious diseases, neutralizing antibody titre is almost universally considered a marker of protective immunity [21, 22], and several studies suggest that this relation may be mechanistic [23]. For instance, the transfer of vaccine-induced serum antibodies between individuals tends to confer short-term protection from infection [24]. In contrast, the wide range of anti-tumour mechanisms employed by the immune system can make identifying immunological surrogates challenging. Significant cancer heterogeneity [25] and the immunosuppressive tumour microenvironment (TME) [26] complicates things further, by contributing to vaccine inefficacy even when ‘positive’ immunological markers may be present. It is therefore unlikely that a single cross-applicable immunological correlate for cancer vaccines exists to the same degree that antibody titre does for infectious disease. Instead, multiple different measures of the immunological response may need to be combined to form reliable immunological surrogates, likely varying by vaccine candidate. In particular, the rise in ‘omic’ methods enables the collection of more comprehensive proteomic, transcriptomic and genomic data that can better capture the immune response. These integrated approaches may help define correlates that more accurately infer vaccine efficacy, despite significant patient heterogeneity, making small immunobridging trials a feasible option.

Most cancer vaccines are usually not applied prophylactically like their infectious disease counterparts, and so immunological surrogates cannot be developed using challenge studies to link immune response to protection from infection. It is envisaged that clinical endpoints evaluated in cancer vaccine trials, such as Overall Survival (OS) or responses evaluated according to iRECIST criteria [27] are likely to be used to link immunological markers to clinical efficacy (Table 2).

**Table 2.** Comparison of immunobridging trials for infectious diseases versus cancer vaccines.

	Infectious disease vaccine	Therapeutic vaccine
Clinical endpoint	Protection (e.g. symptomatic infection, severe disease)	Overall Survival, Disease-Free Survival, Response (Complete Response, Partial Response, Progressive Disease)
Nature of immune response	Primarily humoral	Primarily cellular, but can include humoral components
Patient population	Generally healthy individuals	Patients with existing cancer, often with compromised immune systems
Immunological surrogate	Primarily neutralising antibody titre	Undetermined
Immunological assay	Enzyme-linked immunosorbent assay (ELISA), Haemagglutination inhibition assay (HAI)	Likely multiple assays measuring several dimensions of the immune response
Regulatory considerations	Established pathways for approval and licensure	Emerging pathways, often requiring adaptive trial designs and accelerated approval processes
Likely application of immunobridging	Booster shots to new variants New patient cohorts	Update according to new adjuvants, platforms, and technologies Evaluate combination therapy Dosing optimization
Challenges and considerations	Pathogen mutation, vaccine hesitancy	Tumour heterogeneity, immune evasion, interpatient variability

## Creating immunological surrogates

### Immunoassays

**Figure 3.**
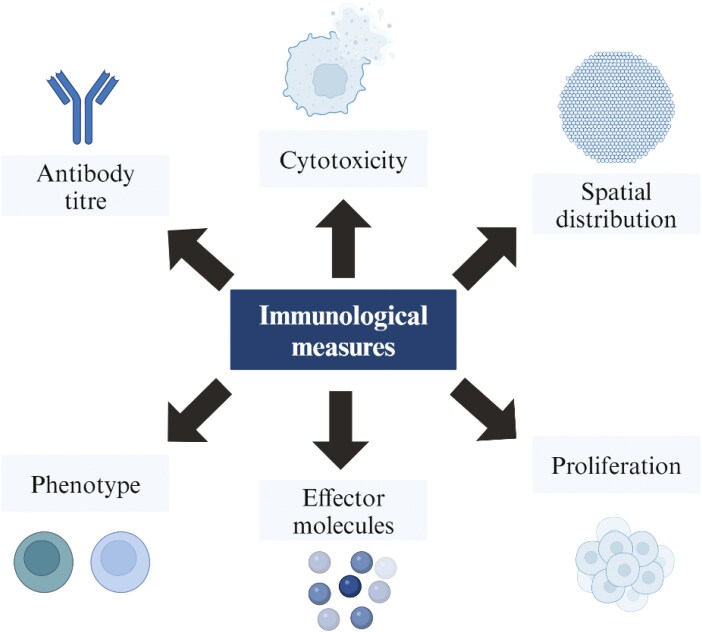
Selection of the different dimensions of the immunological response measured by immunoassays.

Creating reliable and robust immunoassays to measure elements of the cancer vaccine immune response is critical to creating an immunological surrogate. Due to the complexity of the human immune response, a large range of assays can be deployed to measure many different dimensions of both the cell-mediated and humoral response, including antibody titre, proliferation, spatial distribution, phenotype, and functional measures of T-cell response (Fig. 3) [28].

This presents a challenge as even when assays measure the same immunological endpoint, cross-assay standardization using convalescent serum as applied in infectious disease vaccinology is not possible. Chosen immunoassays should be standardized, adhere to International Units (IU) and be kept consistent across different trial phases, locations and research groups so that clinical and immunological data can be pooled to cross-verify and better link surrogates to clinical endpoints [20]. Cancer vaccines often generate highly complex immune responses, and so the requirement of prior immunological knowledge of effector functions or T-cell subtype should be minimized to best capture as many vaccine-specific immunological markers as possible [28]. This point is particularly important, as often correlates of protection are difficult to predict *a priori*. Important examples include the phenomenon of ‘antigen spread’, observed in vaccines like sipuleucel-T where secondary antibodies were found to target prostate cancer-associated tumour antigens distinct from the original PA2024 target antigen [29].

**Figure 4.**
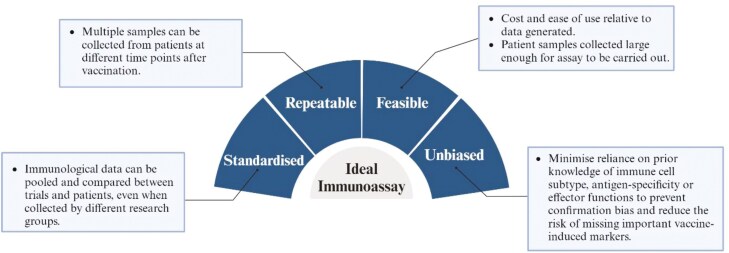
Key considerations in selecting immunological assays for creating surrogates.

Table 3 summarizes the commonly used immunoassays and assesses their applicability for use in creating immunological surrogates based on the criteria outlined (Fig. 4).

**Table 3.** Summary of different immunoassays and their suitability for application in creating immunological surrogates. Key: Highly suitable (++), Suitable (+), Limited suitability (+/-), Unsuitable (-)

Assay	Immune response indicator	Suitability	Justification
ELISA	Antibody titre	+/-	Historically validated and standardized assay that is simple to perform. However, it is only suitable for evaluating peripheral samples and is dependent on antibody titre being a good indication of immunological response.
Chemiluminescence [30]	Antibody titre	+	Chemiluminescence may be preferable to ELISA for quantification of antibody titres due to a wider dynamic range and higher sensitivity. Measures luminescence, which is an absolute measure rather than absorbance which is a relative one, making it easier to cross-standardize data, e.g. between different laboratories and therefore pool clinical data across trials.
Delayed type hypersensitivity (DTH)	Adaptive immune response	--	Cancer vaccines have complex dose-response relationships which makes deciding a standard challenge dose difficult. Difficult to quantify and is inherently variable, making it more suitable as a preliminary screen than a surrogate.
ELISpot	Effector molecules	+	Commonly used robust and standardized assay measures both secreted proteins and number of cells. However, typically measures one effector function at a time and is biased to known effector molecules.
Intracellular cytokine staining [31]	Effector molecules	+	Allows for detection of cytokines at the single-cell level and assessment of multiple cytokines concurrently. Standard ICS without multiplexing tend to require large samples of cells which may not always be accessible.
Multiplex cytokine detection	Effector molecules	+	Suitable for measuring multiple cytokines at the same time to give a more comprehensive overview of cytokine profile. Requires smaller sample volume than ICS, which is helpful for TIL samples where number of cells collected can be limited. However, may be challenging to standardize across centres and results can be difficult to analyse due to cytokine release non-specific to the immune response.
Luminex assay	Effector molecules	+	Allows for multiplexed detection of cytokines and immune-related proteins with higher sensitivity and a more comprehensive panel than ELISA. Could provide detailed understanding of cytokine and chemokine milieu in response to vaccination, identifying key mediators. By measuring multiple analytes simultaneously, provides a more comprehensive view of the immune landscape. However, requires specialist equipment and validated antibody panels, Additionally, the biological relevance of changes in cytokine and chemokine levels requires validation.
Chromium (Cr51) release	Cytotoxic T-lymphocytes	-	Non-quantitative and bulk results which do not provide information on the mechanisms of cytotoxicity. Standardization is difficult, as loading of chromium-51 can differ with respect to individual cells and cell types.
CD107a/b degranulation (flow cytometry)	Cytotoxic T-lymphocytes	+	Measures specific effector function of cytotoxicity, which can then be paired with other assays. However, does not directly measure the killing of cells.
[H3] Thymidine	Proliferation	-	Unstandardized *in vitro* stimulation and expansion protocols making cross-study interpretation challenging. Does not directly measure anti-tumour effector function. Thymidine may interfere with DNA synthesis.
Dye dilution proliferation assays	Proliferation	-	Unstandardized *in vitro* stimulation and expansion protocols making cross-study interpretation challenging. Does not examine anti-tumour effector function. Quantification is less accurate than [H3] thymidine, however, allows single-cell phenotyping.
Ki67 nuclear antigen detection (flow cytometry) [32]	Proliferation	+/-	Avoids *in vitro* stimulation protocols making it easier to standardize between trials and gives a more accurate insight into *in vivo* conditions. However, this method does not capture spatial distribution and is difficult to quantify reliably.
Single-cell RNA sequencing	Functional state	+	High-resolution overview of gene expression, helping identify rare T-cell subtypes and various aspects of the functional state of T-cells. However, can be technically complex and expensive, although it is becoming cheaper to perform.
NanoString	Functional state	+	Profiles gene expression, including immune-related genes, for the immune landscape of the tumour microenvironment. May help identify signatures associated with vaccine efficacy and immune activation. However, limited to a predefined set of genes and lacks single-cell resolution, which may not capture full complexity of immune response. Cost can be high, which may limit widespread use in trials.
ATAC-Seq	Functional state	+/-	Provides genome-wide chromatin data to show epigenetic regulation of immune responses. Useful for the epigenetic landscape of immune cells, identifying signatures associated with vaccine efficacy and immune activation. However, requires specialized sequencing, lacks single-cell resolution, and the biological relevance of epigenetic changes detected may require additional studies to validate.
Single-cell ATAC-seq (scATAC-seq)	Functional state	+/-	Offers high-resolution chromatin accessibility data at the single-cell level, demonstrating heterogeneity in immune cell responses. Can help identify epigenetic signatures associated with vaccine efficacy and immune activation at the individual cell level. However, requires specialized sequencing and the biological relevance likely requires validation through functional studies. Compared to ATAC-seq, scATAC-seq has more limited throughput, which could be a challenge when working with large numbers of samples.
Phosph-flow cytometry	Functional state	+	By measuring phosphorylation state of specific proteins at the single-cell level, provides understanding of immune cell signalling pathways and activation states. Useful for understanding signalling cascades that are activated or inhibited in response to vaccines, potentially identifying key pathways that mediate response. However, requires specific antibodies and specialized equipment. Biological relevance of changes in protein phosphorylation not well characterized. Limited to known signalling pathways and phosphorylation sites, which may not provide a comprehensive view of immune response.
Immunohistochemistry (IHC)	Spatial distribution	-	Cost effective and can provide information on the spatial distribution of T-cells in biopsies. However, interpretation is subjective and staining protocols are not internationally standardized. Spatial data is limited to biopsy sample, which does not reflect the full heterogeneity of the tumour and so can be challenging to standardize between patients when forming a surrogate.
RNA *in situ* hybridization	Spatial distribution	+/-	Visualizes gene expression within the tissue, providing context regarding immune cell distribution and interactions within the tumour microenvironment. May help to identify regions of the tumour more responsive to vaccination. However, limited to a set of target genes and requires specialist equipment and expertise. Moreover, quantification of gene expression can be challenging.
Positron emission tomography (PET) imaging [33]	Spatial distribution	+	Enables measure of T-cell distribution across the tumour *in vivo*, rather than based on small sections. Data are more easily quantified and less subjective. However, requires access to PET scanners and can only evaluate one marker at a time.
Spatial transcriptomics [34]	Spatial distribution	+	Can be used to analyse the TME and pinpoint interaction between tumour cells and immune cells. Paired with gene expression pattern information gives insights on the spatially dependent functional state of T-cells.
Cytometry by time of flight (CYTOF)	Immune markers	++	Measures up to 50 parameters per cell and does not suffer from technical limitations of spectral overlap like flow cytometry methods, although is more expensive and technically demanding.
Multiparameter Flow cytometry	Phenotype	+	Analysis of T-cell subsets, including multiple simultaneous markers. Can measure both effector and inhibitory cells. Can be easily combined with ICS to generate insight into effector function. It is also cheaper and less technically complex to perform then spectral flow cytometry. However, future applicability is limited due to technical limits in differentiating increasingly small T-cell subtypes.
Spectral flow cytometry	Phenotype	+	Measures up to 40 different parameters and is better at analysing multiple markers simultaneously to differentiate rare or undefined T-cell populations.
T-cell homing assay	Phenotype	-	Measure of the ability of T-cells to migrate to and infiltrate tumour sites. Can assess the trafficking potential of vaccine-induced T-cells and identify factors that may improve or inhibit their recruitment to tumours. Valuable for understanding mechanisms that regulate T-cell trafficking and identifying strategies to improve T-cell infiltration in the context of cancer vaccines. However, limited by complexity of the tumour microenvironment, not recapitulated in *in vitro* assays. Additionally, specific chemokines and adhesion molecules involved in T-cell trafficking may vary depending on the tumur type and location, requiring the development of tailored assays. Does not provide information on the functional state or antigen specificity of infiltrating T-cells.
T-cell receptor (TCR) sequencing	T-cell-based	++	Provides comprehensive assessment of T-cell receptor repertoire, allowing for identification of antigen-specific T-cell clones and tracking their expansion in response to cancer vaccines. Useful for understanding clonal composition of T-cell response and identifying dominant T-cell clones associated with vaccine efficacy. However, requires specialist expertise in library preparation and data analysis and the relevance of specific T-cell clones requires validation. TCR sequencing does not provide information on functional state or phenotype of T-cells, which may be important for understanding the overall immune response.
Tetramer staining (flow cytometry)	T-cell-based	+	Allows for direct quantification of antigen-specific T-cells, providing sensitive and specific readout of T-cell response. Using fluorescently labelled MHC-peptide complexes, can identify T-cells that recognize specific tumour antigens, allowing for tracking of antigen-specific T-cell populations over time. Good for monitoring magnitude and durability of T-cell response to and identifying potential correlates of protection. However, requires prior knowledge of the specific MHC-peptide complexes recognized by T-cells, which may limit its applicability. Additionally, sensitivity may be limited in detecting low-frequency antigen-specific T-cells. Does not provide information on the functional state or phenotype of antigen-specific T-cells.
Circulating tumour DNA (ctDNA)	Tumour destruction	+	Offers a non-invasive approach for detecting and monitoring tumour responsiveness to cancer vaccines. However, it faces challenges with standardization and cross-trial application, and its applicability needs further investigation.

Quantifying cell-free circulating tumour DNA (ctDNA) shed into systemic circulation may be a useful biomarker of therapeutic response, which could be used in conjunction with or as an alternative to immunoassays. Collecting ctDNA samples is minimally invasive meaning multiple samples can be taken throughout treatment duration. Additionally, the short half-life of ctDNA enables more granular and immediate real-time assessment of therapeutic response and outcome [35]. As a prognostic tool, ctDNA is already routinely used in advanced malignancies such as non-small cell lung cancer (NSCLC) to aid in treatment selection [36], and there are early signs that ctDNA biomarkers could be used to evaluate the treatment responsiveness of a tumour. For example, 125 patients enrolled in the CAMELLIA study on metastatic breast cancer had ctDNA samples taken to calculate a molecular tumour index (mTBI) based on 1021 genes [37]. Patients with large mTBI decreases throughout the course of treatment were found to be correlated with longer progression-free survival and overall survival. However, ctDNA methodologies face similar challenges with standardization and cross-trial application to immunoassays, and it is still unclear whether they can be reliably used in the context of cancer vaccines. Further research is likely needed to understand the sensitivity of ctDNA methods in discerning the cell-mediated immune response of cancer vaccines.

### Humoral response

**Figure 5.**
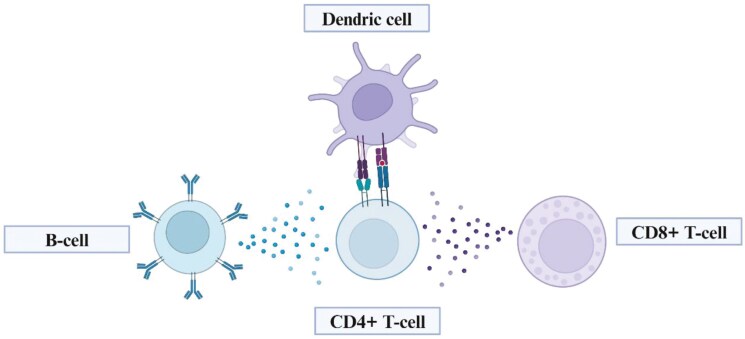
Overlap between CD8+ and B-cell activation: Although B-cell activation may not have a direct anti-tumour effect, it may serve as a reliable correlate for the activation of the underlying T-cell repertoire.

Antibody titre is commonly measured in cancer vaccine clinical trials, typically via enzyme-linked lmmunosorbent assay (ELISA) [20]. Although cancer vaccines would ideally seek to elicit a cytotoxic T-cell response, measuring cancer-antigen-specific antibody release can act as a marker for effective immune response due to overlap with the underlying CD4+ repertoire needed for activating cytotoxic CD8+ T-cells (Fig. 5). For instance, in BALB/c mice vaccinated with autophagosome-enriched vaccines derived from 4T1 mammary carcinoma, antigen-specific IgG and CD8+ T-cell responses were correlated with each other [38], and several clinical trials have linked serum antibody titre with clinical endpoints [39]. However, the extent to which antibody release is a mechanistic or non-redundant correlate of anti-tumour activity is difficult to fully determine, due to the large number of antibody-dependent and independent anti-tumour mechanisms, as well as the inherent overlap and cross-signalling between T and B-cells [40].

However, B-cell depletion studies in mice [41] and the anti-tumour activity of direct cross-injection of natural IgG antibody between cancer patients [42] indicate the capacity for the humoral response to mediate a direct anti-tumour effect. The extent to which antibody titre is a *mechanistic* surrogate for the clinical efficacy of a vaccine candidate is likely influenced by the choice of vaccine platform. For instance, vaccines that preferentially activate the CD8+ T-cell response, such as through neoantigens that are selectively presented on MHC class I, tend to bypass CD4+ activation [43] and therefore may not sufficiently activate antibody-producing B-cells. Measuring other aspects of the B-cell response beyond antibody titre such as the presence of activated B-cells at the tumour site [44], which can mediate a direct killing effect may also form an important part of the vaccine-induced immune response, however, are rarely evaluated in most cancer vaccine trials. Further studies are needed to elucidate and isolate elements of the humoral response mechanistically associated with anti-tumour effects so that more appropriate assays can be selected.

### Cell-mediated response

Cell-mediated assays are used to assess various elements of the T-cell response. In particular, the function and presence of antigen-specific CD4+ and CD8+ T-cells are commonly evaluated, typically via IFN-gamma ELISpot, proliferation assays and flow cytometry subset analysis [20]. However, although some clinical trials have found associations between T-cell and clinical response using these standard assays, many trials have failed to do so. A factor that may be driving this high failure rate is the use of peripheral rather than tumour-infiltrating T-lymphocyte (TIL) samples, which can differ significantly in character and composition. For example, in phase II clinical trials of FDA-approved Sipuleucel-T, significant increases in activity were observed in multiple T-cell subtypes of infiltrating immune cells [45], however, these changes did not correlate with changes observed in peripheral samples. IL-2 expanded TILs have also been associated with higher levels of lung and liver tumour regression in mice compared to peripheral T-lymphocytes, indicating a closer association with anti-tumour activity [46], although mouse models do not perfectly encapsulate the human immune response.

The location of this immune response may also be relevant to certain subsets of immune cells associated with anti-tumour activity, such as CD8+ CD103+ T-cells, which rarely circulate in the periphery [47]. Activated T-cells in the periphery must also undergo infiltration of the tumour to mediate most anti-tumour effects [48]. Isolated immune cells may therefore demonstrate anti-tumour activity *in vitro* but can be inhibited *in vivo* by TME-associated immunosuppressive lymphocytes, immunological checkpoints, and protection from infiltration by stromal cells. Additionally, the distribution of TILs *within* certain tumour types may also be linked to positive clinical outcomes and could form the basis of mechanistic immunological surrogates. For instance, in TNBC tumours, a uniformly high TIL infiltration is linked to better disease-free survival (DFS) [49], however, this association breaks down in the case of heterogenous or uniformly low TIL infiltration. As a result, evaluating TILs is likely to yield a more accurate immunological surrogate for vaccine efficacy.

Despite these advantages, obtaining TIL samples faces numerous challenges, including the need for multiple core needle biopsies [50] which are more suitable for use in accessible tumours such as breast cancers. In addition, the success rate of obtaining paired evaluable tumour biopsies from patients in clinical trials is around 70%, in part due to the risk of iatrogenic complication [51]. This means trials must account for lower amounts of immunological data, which can be challenging when most phase I/II cancer vaccine trials have few responders. There is also a significant degree of spatial heterogeneity of TILs in the TME [52], which could mean differences observed between biopsies are falsely attributed to the treatment effect. This can be circumvented by collecting multiple samples across the tumour and repeating samples in the same tumour locations to facilitate comparison. Multiple biopsy readings can then be analysed with deep learning tools that can quantify TIL densities and potentially better capture TIL heterogeneity [53], although the risk of taking multiple biopsies altering the TME should be considered. Some tumours, such as pancreatic adenocarcinomas [54], are particularly challenging to access for biopsy, while others, such as glioblastomas, are often unsuitable for routine sampling in clinical trials. In these instances, Peripheral Blood Mononuclear Cells (PBMC) samples provide a practical alternative, allowing for continuous immune monitoring due to being less invasive. This approach offers a more dynamic perspective on immune responses as they evolve over time. However, PBMCs are less likely to capture tumour-specific mechanistic correlates compared to TILs and may therefore yield weaker correlates [55]. Combining PBMC analysis with selective TIL biopsies, where feasible, may help balance the need for practicality and mechanistic relevance, for a more comprehensive understanding of immune responses in cancer vaccine trials.

Alternatively, novel T-cell-specific PET imaging modalities, which can use marker-specific radiotracers to map the distribution, abundance and functionality of T-cells in real time may better evaluate TILs [33] by overcoming the subjectivity and restricted reproducibility of standard immunohistochemistry measures using single or multiple biopsies. A better understanding of the spatial distribution of TILs is essential in uncovering spatially dependent immunological correlates of vaccine efficacy. These are likely to exist, with a recent meta-analysis showing that high CD3+ TIL infiltrate in the invasive tumour margins was predictive of OS in colorectal cancer [56], but that this association broke down when compared to CD3+ levels in the stroma or centre of the tumour.

Large meta-analyses collect huge amounts of immunological data to aid in predicting cancer prognosis and patient responsiveness to treatment. Whilst not explicitly studying immunological response to cancer vaccine, these analyses pool large amounts of immunological data and link them to clinical endpoints and so could be indicative of potential correlates of vaccine efficacy. For instance, a recent meta-analysis [57] indicated that a high CD8+/FoxP3+ ratio was predictive of OS, and this was similarly observed in both mouse models and patients receiving G-VAX [58], a GM-CSF gene-transfected tumour cell vaccine. However, whilst these markers may provide a good starting point for determining correlates, they should be cross applied with caution as they are not necessarily mechanistically involved in the anti-tumour effect. Additionally, cancer vaccines may employ different immunological effector mechanisms that are not captured by these predictive studies. Immunological markers may be confounded with a patient’s overall health condition [59, 60], with healthier patients more likely to respond to treatment. Predictive markers that are associated with lower tumour recurrence may partially overcome some of these issues and be a more reliable way to infer markers correlated with anti-tumour activity based on these studies.

Measurement of inhibitory immunological mechanisms is often overlooked in cancer vaccine trials but may act as useful immunological surrogates for indicating a lack of response to a therapeutic vaccine. For instance, immunization of melanoma patients with MHC class I restricted peptides or tumour lysate-loaded APCs induced a significant increase in antigen-specific CD8+ T-cells, yet these responses rapidly decreased back to baseline levels, found to be brought about by rapid Treg expansion [61]. The diversity of both inhibitory and cytotoxic T-cell responses, along with the heterogeneity in their subtypes and spatial distribution, highlights the need for a standardized scoring system that integrates multiple immune parameters to better capture the distribution and variety of T-cell subtypes involved in a successful immune response. For example, an ‘immunoscore’ can be developed to quantify different lymphocyte populations and their densities within TIL samples, potentially providing a more refined measure of immune activity. Immunoscores have already been validated as predictive-prognostic tools in colorectal cancer, where they incorporate CD3+ and CD8+ T-cell densities [62]. A similar approach could be adapted for other cancers using different sampling techniques and immunoassays. While TIL-based immunoscores may offer a closer reflection of intratumoral immune activity, PBMC-based immunoscores could provide a more practical, minimally invasive alternative, enabling serial monitoring of immune responses in clinical trials. Also, depending on feasibility, emerging spatial transcriptomics or high-dimensional imaging techniques could further refine immunoscores by capturing spatial relationships between immune cells within the tumour microenvironment, adding an extra layer of predictive value.

Another significant barrier to the identification of cell-mediated immunological correlates is the significant complexity and difficulty of predicting the cell-mediated immune response. This reflects a fundamental issue in taking a narrow approach of measuring a few potential surrogates such as antibodies or T-cells specific to a TAA used as the basis of a vaccine candidate [20]. These approaches, whilst theoretically justified, often fail to reliably capture the complex realities of the vaccine-induced response and thus generate surrogates. First, many cancer vaccine candidates will not have clearly defined TAA or TSA targets at the outset. These include personalized neoantigen, whole tumour cell, and certain dendritic cell vaccines. Additionally, even when the target antigen is known, a successful anti-tumour response may not necessarily be driven by primary antibodies against the target [63]. For instance, in clinical trials for Sipuleucel-T^29^, the presence of secondary antibodies against non-targeted tumour antigens was associated with improved overall survival. This suggests that antigen spreading may contribute to broader immune activation beyond the initially targeted antigens. This is further compounded by tumour heterogeneity, where the diverse genetic and molecular characteristics of different regions of a tumour make it challenging to identify a single set of immunological markers that accurately reflect the entire tumour burden [64]. Given this, tracking antigen spreading in clinical trials requires integrating high-resolution immune monitoring methods with longitudinal sampling strategies to assess vaccine-induced immune responses in a more comprehensive manner. Future studies incorporating multi-omic and real-time tracking approaches will be needed to refine our understanding of antigen spreading and its clinical significance.

Taking a multi-omics approach to the analysis of the immune response to cancer vaccination is likely to be the best means to capture both tumour heterogeneity and form reliable surrogates. These methods reduce the reliance on studying known immune biomarkers, which have failed to consistently correlate with clinical response in most vaccine candidates. Single-cell RNA sequencing has already successfully been used to identify factors associated with responsiveness to checkpoint inhibition [65] and can be paired with spatial transcriptomics, to associate single-cell data with the structure of the TME [66]. However, challenges remain with these approaches, particularly their high resource demands and the complexity of integrating multi-omic datasets [67]. While decreasing costs and improving methodologies may enable broader use in the future, routine implementation for all patients in each trial is currently impractical. Instead, multi-omic techniques should form the basis of early exploratory analysis in smaller cohorts of patients to identify and narrow down the number of potential biomarkers, which can then be validated using more targeted assays. RNA-seq, in particular, enables a more holistic understanding both in the identification of differentially expressed genes [68], but can also infer T-cell receptor (TCR) diversity [69], antigen presentation [70] and T-cell activation through immune response signatures [71]. Emerging technologies like Nanopore sequencing offer real-time immune profiling capabilities and may help refine adaptive immune response metrics, at an increasingly lower cost [72, 73] Importantly, multi-omic techniques have the potential to overcome the fundamental complexity that has so far limited the ability to link cellular immune responses to protective immunity, by measuring as exhaustively as possible a wide range of different dimensions of the immune response to build a more complete picture and best uncover surrogates whilst minimizing reliance on assumed prior knowledge.

Applying these techniques can also enable a more holistic understanding of the immune response beyond the analysis of T-cells. For instance, dendritic cells, although a relatively rare immune cell population, are important for initiating antigen-specific T-cell responses against tumours [74]. Their activation status may serve as an important correlate of vaccine efficacy, as seen in the Sipuleucel-T trial, where antigen spreading was observed [29]. Identifying dendritic cell activation signatures in post-vaccination analyses could provide early insights into this process, offering new immune correlates of protection [75]. Similarly, natural killer cells have been associated with both direct tumour destruction and the activation of dendritic cells. Although these cells exist in smaller subpopulations, gene signatures and multi-omic approaches can aid in their identification and categorization [76]. There is growing evidence that indicates that dendritic cells and NK cells may play important mechanistic roles in the anti-tumour response and have been correlated with positive tumour prognosis [74]. To capture these dynamics, holistic multi-omic techniques, including single-cell transcriptomics and proteomics can be leveraged to capture the diversity and function of these cell types. In addition, incorporating such findings into a combined measure like an immunoscore could allow for a more integrative assessment of vaccine-induced immune responses, capturing a more complete picture beyond the conventional T-cell and B-cell framework.

## Considerations for trial design

Appropriate clinical trial design is important for developing and validating immunological surrogates and must be considered from the earliest stages in the vaccine development process. Given the generally low toxicity profiles of most cancer vaccines [77], adverse effects are usually rare, prompting experts to recommend combining phase I and II trials [78]. This approach offers several advantages, including increased statistical power and a larger number of samples analysed. However, despite the potential benefits of combining trials, some early-phase cancer vaccine trials continue to follow the traditional paradigm of separate phase I and II trials. Even when using combined trials, many recruit too few patients to match clinical and immunological data or even observe clinical responders (Table 4).

**Table 4.** Summary of recent cancer vaccine trials and barriers in their design to formulating surrogates.

Study	Background	Immune monitoring	Limitations
Youn et al. (2020) [79]	Phase II trial of GX-188E and pembrolizumab combination therapy in patients with recurrent or advanced inoperable cervical cancer.	IFN-gamma ELISpot performed on peripheral blood mononuclear cell (PBMC) samples of all patients. Subgroup of patients with E6 and E7 specific T-cells failed to show clinical benefit.	Trial did not evaluate polyfunctional T-cell response, although trials of GX-188E in pre-cancerous CIN3 patients indicated a correlation with lesion regression, so this correlate could not be validated. Only one assay used to measure one immunological marker. No further investigation of patients with positive immunological response but no clinical benefit was performed.
Gatti-Mays et al. (2020) [80]	Phase I trial of concurrent administration of ETBX-011, ETBX-051 and ETBX-061 in patients with advanced colorectal or cholangiocarcinoma	Antigen-specific T-cell response evaluated by measuring absolute number of CD4+ and CD8+ T-cells producing cytokine or positive for CD107a.	Trial designed as pshase I rather than phase I/II trial. Small sample size meant no responders were observed so successful immune response could not be identified. Only peripheral blood lymphocytes were characterized.
DeMaria et al. (2021) [81]	Phase I trial of intravenous administration of MVA-BN-brachyury-TRICOM vaccine in patients with advanced solid tumour enriched with chordoma.	Antigen-specific T-cell response evaluated by measuring absolute number of CD4+ and CD8+ T-cells producing cytokine or positive for CD107a. One patient underwent biopsy to evaluate T-cell infiltrate.	Only single partial-responder underwent further investigation via biopsy, so no comparison of T-cell infiltrate with non-responders to better differentiate surrogates that might be associated with clinical efficacy. Peripheral brachyury-specific T-cells produced in response to the vaccine were transient, suggesting that treatment effect was mediated by degree of infiltration of brachyury-specific T-cells, yet infiltration for most patients was not evaluated.
Morse et al. (2020) [82]	Phase I trial of Vvax001 in patients with CIN3.	IFN-gamma ELISpot responses to E6 and E7 antigens.	No long-term follow-up of participants or evaluation of clinical response. Only peripheral blood lymphocytes evaluated.
Schuhmacher et al. (2020) [83]	Phase I/II trial of synthetic long-peptide vaccine targeting RhoC in patients with prostate cancer.	IFN-gamma ELISpot and intracellular cytokine staining. Subset of patients also had Tregs stained.	Trial was progressed based purely on immunological data and without any patients indicating response to treatment. Follow-on phase II trial found no clinical benefit versus placebo. Likely this is because whilst polyfunctional CD4+ T-cells were observed, few patients displayed antigen-specific CD8+ T-cells. Both signals are likely to be needed for an anti-tumour response.
Crosby et al. (2020) [84]	Phase I trial of VRP-CEA in patients with stage III and IV colorectal cancer.	Stage III patients underwent CYTOF flow cytometry analysis and a small subset had antigen-specific T-cell response evaluate via IFN-gamma ELISPot. Stage IV patients were evaluated only by IFN-gamma ELISpot.	Different immunological assays were performed on stage III and IV colorectal cancer patients limiting cross-comparison between groups.

Another aspect of clinical trial design is the collection of immunological data. In many cases, the methods used to gather immunological data are not well-suited for the creation of surrogates. For example, some trials may only collect baseline patient immunological data, making it difficult to assess the vaccine-induced immune response over time. Additionally, the use of different assays between patients or trials can complicate data interpretation and limit the ability to compare results across studies [28]. Finally, relying on immunoassays that lack sufficient sensitivity or only measure a single aspect of the immune response may fail to capture the full complexity of vaccine-induced immunity [5]. To address these challenges, it is important to formulate a set of standardized assays that characterize a broad set of immune functions. These assays should be used consistently in the exploratory characterization of the vaccine-induced immune response during early clinical trials [85]. By adopting a standardized approach, researchers can generate more applicable and comparable immunological data, increasing the likelihood of identifying reliable immunological surrogates of vaccine efficacy.

Clinical trials on patients in a pre-malignant setting or with pre-cancerous lesions may provide an alternative approach to identifying immunological correlates of protection for cancer vaccines. These trials offer the advantage of studying vaccine-induced immune responses in a more homogenous patient population, as these patients generally have fewer confounding factors that may influence the vaccine-induced immune response, such as prior treatment with chemotherapy or immunosuppression associated with advanced tumours [26]. This could allow for a clearer understanding of the relationship between immune responses and clinical outcomes. For example, patients with cervical intraepithelial neoplasia (CIN3) and those with advanced cervical cancer who were treated with the DNA vaccine GX-188E generated E6 and E7 antigen-specific T-cell responses associated with clinical benefit and lesional regression [86]. Similar methodologies could be applied to patients identified as high-risk, such as those with pancreatic lesions, where immunological data could be correlated with a reduced risk of progression to invasive cancer [87]. Findings from such trials could then be validated in studies on advanced tumours, informing vaccine development and optimization.

However, it is important to acknowledge that immunological surrogates identified in pre-cancerous lesions may not fully translate to the advanced cancer setting. One key limitation is that a lower tumour burden in pre-malignant conditions will result in lower antigenic load, potentially influencing the magnitude and quality of vaccine-induced immune responses [88]. While GX-188E successfully elicited E6 and E7 antigen-specific T-cell responses in patients with advanced cervical cancer, a subset of these patients failed to derive clinical benefit despite having high levels of tumour-specific T-cells [79]. This suggests that tumour heterogeneity and the immunosuppressive tumour microenvironment complicate the association between a ‘positive’ immune response and clinical benefit.

Another challenge is the availability of suitable tissue for immune monitoring. While PBMC samples may be the most accessible option in pre-malignant lesion trials, their ability to reflect the tumour microenvironment in advanced cancer is uncertain. In some cases, analysing TILs may provide a more accurate reflection of the local immune response. However, obtaining TIL samples is often more feasible in advanced cancers than in small or inaccessible pre-malignant lesions, limiting their use in early-stage trials. This approach is best suited for cancers where a well-defined pre-malignant stage exists, and antigen expression profiles between the pre-cancerous and malignant states show significant overlap. For example, high-risk pancreatic lesions and HPV-associated cervical intraepithelial neoplasia are strong candidates for this strategy. Conversely, cancers such as small-cell lung cancer, which lack a detectable benign precursor, or colorectal adenomas [89], which have antigenic profiles distinct from colorectal carcinoma, may not be suitable for this approach. Despite these challenges, focusing on pre-malignant lesions remains a valuable strategy for identifying potential immunological correlates, informing the selection of combination therapies, and providing early indicators of vaccine efficacy. This could aid the progression to larger, more resource-intensive clinical trials in patients with advanced disease, accelerating the development and optimization of therapeutic cancer vaccines.

**Figure 6.**
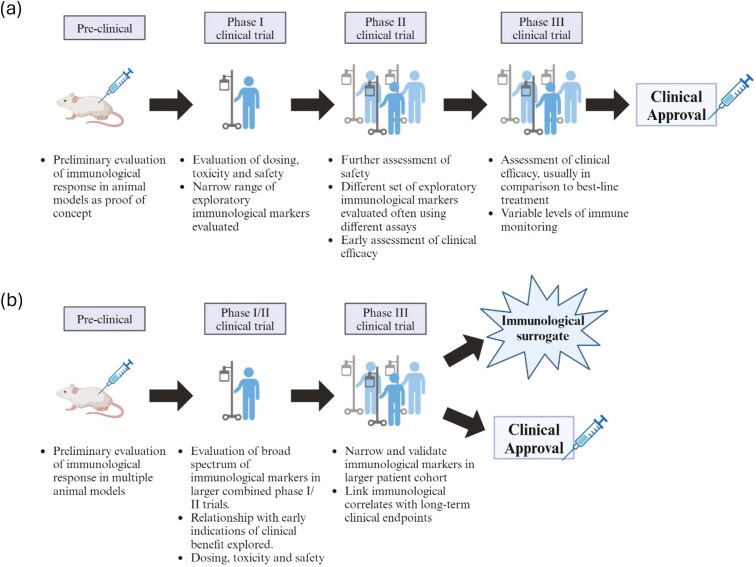
Summary of cancer vaccine development pipeline and adaptations to uncover immunological surrogates. (a) Traditional cancer vaccine development pathway, (b) modified cancer vaccine development pathway, with the goal of formulating an immunological surrogate alongside achieving clinical approval.

In addition to combining phase I and II trials, cancer vaccine development should be adapted for the creation of surrogates at each stage of the development process (Fig. 6). Pre-clinical studies, typically performed in animal models, can help give an early indication of immunological responses associated with desired clinical outcomes [90]. These responses can then be validated in phase I/II trials, where a broad spectrum of immunological markers can be measured. Markers that are believed to be associated with clinical endpoints can then be refined and validated in larger phase III trials, enabling a link to be drawn between certain immunological measures with clinical efficacy and thus form a validated immunological surrogate that ideally could be applied in later clinical trials or follow-on studies involving the same vaccine candidate. Whilst we expect these surrogates to be applied in immunobridging trials for their respective licenced vaccines, it remains uncertain whether surrogates validated in this way can be reliably cross applied to other cancer vaccine candidates that are believed to share similar fundamental mechanisms of action (e.g. mRNA vaccines) or across different tumour types. Early evidence indicates that when the same vaccine candidate successfully elicits a clinical response in multiple tumour types, similar immunological responses are observed [91]. However, whether these responses are truly mechanistic or simply correlate with immune activation without direct clinical benefit remains to be determined.

Due to budgetary or logistical constraints, it may not always be possible to run the same assays or to evaluate the immunological response of patients during clinical trials. In these cases, the long-term storage of patient samples, such as through cryo-preservation, can enable retrospective analysis. Although long-term storage is associated with some loss in viability, particularly of more delicate subsets such as polymorphonuclear leukocytes, TILs can still generally be recovered and evaluated [92], allowing immunological correlates to be formed retrospectively. This provides the advantage of enabling the analysis of old tumour samples with more modern approaches, such as multi-omics assays, allowing new correlates to be identified without having to redo clinical trials. Further research is needed on the length of viability of stored patient tissues and the accuracy of any assays performed on them.

Finally, regulatory authorities need to develop clear frameworks and guidance on the application of immunological surrogates to cancer vaccine development. Currently, although correlates of protection data are used in licencing applications for infectious disease vaccines, they are assessed on a case-by-case basis [11]. Having clear rigorous frameworks and guidelines on the type and strength of evidence needed for approval would help incentivize the development and application of immunoassays to uncover and validate immunological assays in clinical trials.

## Conclusion

Despite the growing promise of cancer vaccines, immunological surrogates of clinical efficacy have yet to be conclusively identified. A lack of consensus on these surrogates may well hinder future opportunities to employ immunobridging, which could be correlated with the clinical effectiveness of cancer vaccines. Several strategies should be prioritized in the near-term to identify immunological correlates of vaccine efficacy. First, trial designs should be optimized to better facilitate surrogate discovery by combining phase I/II trials to better understand early immunological changes. Second, multi-omic approaches that minimize bias need to be systematically employed within and between different trials to more comprehensively profile vaccine-induced responses. Third, standardized protocols, specimen preservation and data sharing across trials would enable the pooling of data to form larger datasets that can then be mined using statistical methods and artificial intelligence to more conclusively link immunological changes to clinical benefit. Finally, regulatory authorities must provide clearer guidance on the evidence required to validate surrogate markers and their use in immunobridging studies to incentivize their uptake.

While these efforts are important, adopting multi-omics approaches such as single-cell sequencing, spatial transcriptomics and high-dimensional cytometry, likely represents the most promising strategy to capture the full complexity and heterogeneity of anti-tumour immune responses. Applying these tools consistently across trials could enable the identification of novel immunological correlates that conventional assays may be unable to detect. The establishment of reliable immunological surrogates as the basis for immunobridging trials has the potential to revolutionize cancer vaccine development. Surrogate-driven approaches could accelerate the optimization of vaccine platforms, adjuvants, combinations and dosing regiments without needing new large-scale phase III trials for each permutation. Efficient trials of vaccines targeting novel tumour antigens would be enabled, expanding access to effective immunotherapies for broader patient populations. In addition, immunobridging would help enable regulatory authorities to meet the challenge of approving AI-based antigen-selection methodologies [93].

In summary, establishing immunological surrogates should be a primary priority for the cancer vaccine field to realize the full potential of this promising therapeutic method. Improving clinical trial designs, clarifying regulatory guidance, standardising key methods, and leveraging multi-omic profiling technologies provide a possible strategic roadmap. As many cancer vaccine trials enter phase II and III clinical trials, now is the opportune moment to collect the requisite data to create validated immunological surrogates to enable immunobridging. While challenges remain, aligning the field around this goal and facilitating close collaboration to systematically identify, validate, and translate robust immunological surrogates into clinical practice is crucial. If successful, this surrogate-driven approach can present an expedient method of optimising cancer vaccines, without compromising on safety or efficacy, saving lives in the process.

## Abbreviations

TAA: Tumour-associated antigen
TSA: Tumour-specific antigen
CoP: Correlate of protection
TME: Tumour microenvironment
OS: Overall survival
IU: International units
ctDNA: Circulating tumour DNA
NSCLC: Non-small-cell lung cancer
mTBI: Molecular Tumour Index
ELISA: Enzyme-linked immunosorbent assay
TIL: Tumour infiltration T-lymphocyte
DFS: Disease-free survival
PBMC: Peripheral blood mononuclear cells
APC: Antigen presenting cell
TCR: T-cell receptor
PMN: Polymorphonuclear leukocytes
